# Continuation of an eating disorders day programme during the COVID-19 pandemic

**DOI:** 10.1186/s40337-021-00390-z

**Published:** 2021-03-09

**Authors:** Sarah Plumley, Anna Kristensen, Paul E. Jenkins

**Affiliations:** 1 Eating Disorders Unit (Block 22), St Mark’s Hospital, 112 St Mark’s Road, Maidenhead, SL6 6DU UK; 2 School of Psychology & Clinical Language Sciences, University of Reading, Harry Pitt Building, Earley Gate, Reading, RG6 6ES UK

**Keywords:** Anorexia nervosa, COVID-19, Partial hospitalisation, Day programme, Virtual treatment, Pandemic, Group therapy

## Abstract

The current paper describes an adaptation of a daypatient programme for adults with anorexia nervosa in the UK in response to the COVID-19 pandemic and consequent government guidelines. The paper details how the programme, which is normally delivered face-to-face, became a ‘virtual’ clinic, providing support to a group of patients via the Internet and conducting its core activities almost exclusively via videoconferencing. Anxiety around the pandemic influenced patients’ feelings about recovery, and there were concerns about the programme moving online, which necessitated careful management. It has been possible to continue an intensive level of care given wider organisational backing and the support of the patients involved. Some of the patients’ reflections on the experience are included in the article. As well as the adaptations, the article also discusses some of the challenges and opportunities encountered, in the hope of guiding similar services.

## Main text

In December 2019, an outbreak of severe acute respiratory syndrome coronavirus 2 (SARS-CoV-2, later designated as COVID-19) was reported in Hubei Province, China. Since then, the world has been grappling with challenges around infection control and limiting the impact of the COVID-19 pandemic [1]. Although resources are understandably directed to those infected and frontline responders, the voices of vulnerable populations, such as people scheduled for elective operations or those with mental health problems, could go unheard, potentially serving to “augment existing health inequalities” [2].

Mental health services have had to adapt to the requirements of safe practice in response to the pandemic [1]. Within the field of eating disorders (EDs), experts have suggested ways that evidence-based treatments can be delivered without the need for face-to-face contact [3, 4], as well as highlighting the unique needs of individuals with EDs during the pandemic [5, 6]. Continuing to provide access to evidence-based care is vital as early studies suggest that many individuals have experienced a worsening of ED symptoms alongside greater anxiety in the early stages of the pandemic [7]. Individuals with restricting EDs have reported increased dietary restriction and individuals with binge-eating problems have reported increases in the frequency of binge eating and urges to binge [8, 9]. Those at highest medical risk often provoke the most concern, and the viability of keeping intensive treatment (such as hospital inpatient and daypatient programmes) running has been highlighted in the earliest papers on COVID-19 and EDs [5, 10, 11].

The current report describes continuation of an established daypatient programme for patients with anorexia nervosa during the COVID-19 pandemic. Specifically, the report details how the intensive treatment programme was adapted to continue the provision of care online, while the challenges met, and those still faced, are also discussed.

### Setting

The Berkshire Eating Disorders Service (BEDS) is a regional service in the south-east of England offering a daypatient (also known as partial hospitalisation) programme and outpatient treatment to individuals and their families. It covers a population of around 900,000 and received around 420 referrals in 2019. The daypatient programme offers intensive treatment for adults with anorexia nervosa and operates 4 days a week. Patients in the day programme need intensive treatment but are functioning sufficiently well that more intensive (inpatient) care is not deemed necessary. All attend the programme voluntarily and express a degree of motivation to recover such that they can manage the demands of daypatient treatment.

Table 1 summarises demographic information and diagnostic characteristics of the nine patients who attended the day programme when it moved online. All met criteria for anorexia nervosa [12], with one individual reporting regular binge eating and purging. Most were White-British and female and this small sample is reflective of a UK sample of day- and inpatients enrolled in a large multi-cohort study [13]. Three individuals had received previous treatment – either psychological therapy, other outpatient care, or inpatient care. Comorbid mental health problems included depression, anxiety, and autism spectrum disorder.

**Table 1:** Characteristics of patients attending the online daypatient programme (*N* = 9)

Gender, n female (%)	8 (88.89%)
Ethnicity, n White-British (%)	7 (77.78%)
Age, years (SD)	30.33 (13.93)
Diagnosis and severity (DSM-5)	
AN-R^a^ – Mild	2 (22.22%)
AN-R – Moderate	1 (11.11%)
AN-R – Severe	2 (22.22%)
AN-R – Extreme	3 (33.33%)
AN-BP^b^ – Moderate	1 (11.11%)
Duration of illness, years (SD)	5.89 (5.41)
Previous treatment (%)	3 (33.33%)
Comorbidity (%)	9 (100.00%)
Employment status	
Employed	4 (44.44%)
Full-time education	4 (44.44%)
Unemployed	1 (11.11%)
Living arrangements	
Alone	1 (11.11%)
With family or partner	8 (88.89%)

^a^Anorexia nervosa (restricting subtype)

^b^Anorexia nervosa (binge-purge subtype)

### Treatment

In common with many daypatient EDs programmes [14], treatment comprises a range of interventions, including therapeutic groups, meal support, and individual support. Adjunctive individual or family therapy is offered in many cases. The daypatient programme is behavioural in ethos and delivery and is underpinned by an integrative psychological model including Cognitive Behaviour Therapy (CBT) and a strong values-based approach, taken from Acceptance and Commitment Therapy. The programme runs in 12-week treatment ‘blocks’, separated by a one-week break where the previous treatment block is evaluated and adjusted according to the needs of the patient group. Typically, a patient will remain in the programme for around three treatment blocks (i.e., 9 months), often reducing their time in the programme in the later part of treatment and engaging more in the community. The daypatient programme can accommodate 8–10 patients at any one time and operates from Monday to Friday, between the hours of 9 am and 2 pm. When the programme initially went ‘virtual’ due to the pandemic, nine patients were attending, with a further five on a waiting list.

### Initial reaction

Given the rapid spread of the pandemic and subsequent changes in national policy, it was initially considered that the programme would have to close, and only resume once safe to do so. However, the risks of doing so, including further isolation and psychological consequences [15, 16], posed additional health risks.

Before strict government guidance was introduced, groups were tailored to address the patients’ fears and to support them in dealing with the incoming restrictions. Issues such as how to adjust their eating when access to some staple items may be restricted due to panic buying and how to structure increased time at home were covered. Patients were also given opportunity to talk through their anxieties and supported to make action plans as much as possible.

A daily group ‘check-in’ using videoconferencing, alongside individual remote monitoring, was initially offered, and there was a focus on how patients could adjust their eating and structure their time at home to cope with the crisis. However, it soon emerged that it might be possible to deliver much of the daypatient programme ‘remotely’ via videoconferencing and e-mail contact.

When it was first suggested that the programme would move online, some patients voiced a concern that the progress they had made so far in treatment would be ‘lost’, an anxiety which was easy to understand. The clinical team insisted from the outset of the change that the expectations of working towards recovery remained as important as ever and encouraged patients to see this as an opportunity to continue, or even accelerate, their progress. Patients were reminded that the goal of daypatient treatment is to translate progress in the programme to progress ‘at home’, and they were assured that they would be supported as fully as possible to achieve this. In line with the values-based approach, patients were encouraged to visualise their recovery – during the COVID-19 crisis and beyond.

### Getting started with videoconferencing

The NHS Trust in which the service operates (Berkshire Healthcare NHS Foundation Trust) arranged for appropriate technology to be provided for maintaining remote contact with current patients. The service was equipped to use Microsoft Teams as the medium for videoconferencing. During group video calls, staff and patients are able see multiple participants at once, write messages in a meeting ‘chat’, share content from their desktop, turn their cameras on and off, blur their background, and mute and unmute themselves (and others). Risks relating to data protection due to using online technology were acknowledged and all patients accepted this condition and gave written consent for their email addresses to be shared with other group members. Staff members were reminded to be mindful around confidentiality issues related to the increased use of emails. Practically, videoconferencing was an available option as staff could use their work laptops at home, and all patients had access to computers or smartphones. It should be acknowledged that issues such as Internet access, visual impairments, and so on, are important considerations when moving to an online programme.

### Therapeutic groups

Most of the therapeutic groups of the daypatient programme were continued over videoconferencing, with patients and staff ‘attending’ twice a day for the hour-long sessions. The ethos throughout has been that the clinical team and patients work together to find ways to adapt. It has been found that some groups lend themselves to virtual delivery more easily than others. For example, CBT groups with a clear structure and activities (e.g., worksheets) that can be completed during the session have been straightforward to facilitate. Creative groups and those requiring a higher level of patient interaction have been more of a challenge. Groups looking at particularly emotive topics (e.g., relationships, body image) have been challenging to manage in a virtual setting, but these have continued with additional reminders to patients that they can reach out to staff if they need to.

Videoconferencing can hinder free-flowing dialogue and the nuances of group dynamics (such as body language, group member interactions, and therapeutic silence) can be more difficult to assess over video [17], which has necessitated particular attention and flexibility. In line with wider research [17], the team has found that groups work best where the facilitator makes the objectives of the session clear and takes control in leading the session, for example, by inviting contributions from group members in turn rather than leaving it open to all talking at once – or not at all. Group facilitators have also found that it has helped to set an activity during the session that can be completed individually and then reflected on as a group. Resources for such activities have on these occasions been emailed to patients prior to the session. The ‘chat’ function has been activated for group members seeking clarity on any issue raised in session and likewise group co-facilitators have been able to use this function to reinforce learning points during session.

### Meal support

Meal support forms the foundation of the daypatient programme given the key tenets of behavioural approaches and the importance of normalising eating behaviours [18]. Meal support is prioritised above other activities, which are delivered around ‘protected’ mealtimes. Given the severity of the patients’ illnesses, and the fact that many are unable to restore weight with less intensive treatment, the presence of this support is critical. However, meal support via videoconferencing presented the biggest challenge to the continuation of care, and it remains somewhat challenging to ensure patients complete meals.

At meal support times patients and staff join online. The virtual programme follows the same meal timings and same meal plan as the face-to-face programme. Patients have been asked to position their camera to allow staff to see them eating and what is on their plate. The opportunity to omit or exchange items of the meal plan is made easier in virtual meal support than face-to-face and therefore this intervention relies on astute clinical observation from the staff member as well as honesty on behalf of the patient. If a patient wishes to use the opportunity to avoid adherence to the meal plan or exchange an item for a lower calorie option, then virtual working does afford this.

Virtual meal support is unlikely to have any meaningful impact if a patient is resistant to change. As with the face-to-face day programme staff are unable to verify with surety what is being eaten at every meal. Like with the face-to-face programme, compliance with meal plans is evaluated through self-monitoring forms alongside evidence of behavioural change and, in turn, weight restoration. An additional issue is that of attendance. Due to the new home dynamics caused by the pandemic, it appears that some patients prioritise meals with their family or may need to attend to their children at mealtimes. This is unfortunate for those where the programme’s meal support would have resulted in better adherence to the meal plan. Staff have reminded patients to attend meal support if they are not managing to follow the meal plan, but flexibility has also been necessary to allow these new commitments, such as childcare.

### Regular weighing

Ordinarily a patient is expected to gain a minimum of 0.5 kg a week in the day programme. In acknowledgment of the increased stress of the pandemic for patients as well as reduction in support available the weight expectation at the start of virtual treatment was adjusted. The Service retained the emphasis on weight recovery but removed the boundary of 0.5 kg, accepting any increment of weight increase each week. Prior to the pandemic, daypatients were weighed once a week in the programme in line with typical CBT protocols [19, 20]. As part of virtual delivery of the programme, patients either weigh themselves alone once a week at their residence or ask someone in their household to accompany them and record the weight. Patients are then asked to e-mail the clinical team with their weight on a Monday morning; however, weighing has been a challenge for a small minority of patients who do not wish to have scales in their home due to concerns that this would influence their ED negatively. Like adherence to the meal plan, the accuracy of a patient’s weight relies on the patient’s honesty. There has, therefore, been an increased need for clinicians to evaluate patients’ physical health based on contact during videoconferencing, even if this evaluation is of limited accuracy. At times, verification of a patient’s accurate weight has been sought through a family member, GP, or in a one-off visit to the clinic in person. Any specific issue regarding weight progress is discussed with the patient during the week and an action plan agreed in individual support sessions.

### Individual support

Each patient has a designated keyworker throughout their time in the programme. Keyworkers meet individually with patients to go through self-monitoring records, review risk, and ensure that the patient is continuing to make progress in line with their goals. These sessions have remained largely similar to the face-to-face sessions, as these are one-to-one conversations. Patients in the programme also have access to individual therapy which continues remotely, unless a patient prefers to suspend this until face-to-face treatment can resume [4]. While research on videoconferencing therapy suggests that the therapeutic alliance can be formed, and good patient outcomes achieved [21], some patients nevertheless preferred waiting for face-to-face therapy. Finally, individual medical management continued over telephone consultations, with some tasks such as blood testing, being delegated to the patient’s primary care physician to avoid unnecessary travel.

### Admitting and discharging patients

During the online adaptation of the daypatient programme, patients have been discharged from the programme, and other patients have been welcomed. The patients who were discharged demonstrate the relatively successful support of the virtual programme during this otherwise stressful time for patients. However, while the components of the programme are running online, new patients who require a lot of input have underlined how this virtual version is not as intensive as the face-to-face alternative. That is, participating in the day programme from home leaves patients alone between meals and therapeutic groups and, as mentioned, meal support has limitations. Nevertheless, the new patients have integrated well into the programme and virtual working has not impacted on assessment and commencement of treatment.

### Clinical outcome data

We report data on this small sample, summarising clinical information at pre- and post-treatment in Table 2. Several self-report measures are administered as part of the programme, including the Eating Disorder Examination Questionnaire (EDE-Q) [23] to assess eating pathology, the 9-item Patient Health questionnaire (PHQ-9) [24] to assess depression, and the 7-item Generalized Anxiety Disorder questionnaire (GAD-7) [25] to assess anxiety. Although limitations should be borne in mind, the findings are in line with previous evaluations of face-to-face approaches [13]. Mean BMI was higher at post-treatment (18.36, SD = 1.71) than pre-treatment (15.38, SD = 1.82), evidencing a large effect size (see Table 2). Improvements were noted for other variables, such as eating psychopathology, depression, and anxiety. Of the nine patients who began daypatient treatment, four completed treatment as planned. The remaining five either declined further daypatient treatment ‘against medical advice’ or transferred their care to other NHS trust eating disorders services due to moving out of area.

**Table 2:** Mean (SD) scores on clinical measures at admission and discharge (*N* = 9)

Measure	Admission	Discharge	Effect size (Hedge’s *g*_*av*_) [22]
BMI, kg/m^2^	15.38 (1.82)	18.36 (1.71)	1.52
EDE-Q Global	4.78 (1.02)	3.26 (1.47)	0.97
EDE-Q Restraint	4.91 (0.77)	2.98 (1.60)	1.39
EDE-Q Eating Concern	4.21 (0.87)	2.71 (1.47)	1.11
EDE-Q Shape Concern	5.15 (1.31)	4.26 (1.66)	0.52
EDE-Q Weight Concern	4.84 (1.61)	3.31 (1.87)	0.79
PHQ-9	20.78 (5.14)	16.22 (7.12)	0.66
GAD-7	15.56 (5.57)	13.67 (4.77)	0.33
Discharge status (N, %)			
Treatment completion	–	4 (44.44%)	–
In other treatment	–	5 (55.56%)	–

### The patient experience

The full picture of the patient experience may only be known once ‘normal’ service has resumed and it is too early to draw any firm conclusions on the programme’s effectiveness. However, some tentative conclusions can be drawn based on the experience of those who attended the programme, and the limited clinical data available. The patient group agreed to provide feedback, excerpts of which are included below.

Patients reported reservations about the programme moving online and that their anxiety increased significantly during this transition – both about the global crisis and the implications for their recovery. As one patient noted, “I felt like my support had been taken away from me at a time in which I was just beginning to make progress and thought that maybe it was some sort of sign that I just wasn’t meant to get well again.”

For some of the group, video communication presents its own anxiety although many have been able to challenge this anxiety and have persevered to maintain a sense of group cohesion. One patient commented that she “decided to try it once, [and] if it was totally awful then I wouldn’t do it again. Myself and a few of the other patients checked in with one another moments before the first session, expressed our anxieties and all decided to ‘grab the bull by the horns’ and do it!” Others noted downsides, including that “you can’t interact with the other patients and staff as you would during normal sessions so you don’t quite get the same open discussions which can be really useful. It’s tricky when you may find yourself upset by something as if you were there you would be able to talk it through more easily, but it’s easy to close down and internalise online.”

Both the clinical team and patients are learning to adapt to the ‘new normal’. As one patient reflected, “As the weeks pass by it’s becoming more ‘normal’ and less scary to log on and interact with everyone. The comfort when you see those familiar faces that are there to listen and help you is like the light at the end of the tunnel shining through, even when you feel it may have long faded”. However, practical challenges have also been noted, such as those with childcare commitments or a variable Internet connection.

Lockdown with a serious ED presents a unique challenge, the like of which many patients and staff have never had to face before. Whilst it is too early to draw any conclusions regarding the impact on recovery, the patients appear grateful that the programme has continued and believe that it is making a difference: “I personally think the pluses outweigh the minuses, I feel sure I would have gone completely backwards if I hadn’t had this on offer to me.”

These perspectives offer encouragement to other services to embrace virtual working, albeit with conditions. One patient reflected that “being active in the daily groups is crucial in my recovery and staying on top of the demon within. To any ED service that hasn’t yet set up a virtual group, I cannot recommend it highly enough. Staying connected and continuing treatment during a global health crisis is vital and is proving to be truly invaluable for not just myself but my fellow patients too.”

### Benefits of online working

In spite of the appalling toll of this pandemic, there are several aspects to this new way of working that may remain in place beyond the current social restrictions. Online working across the whole of the BEDS has afforded greater capacity and accessibility of treatment in a way which might not have been considered otherwise. Continuing to improve efficiency is vital to services, like the current one, which see increasing demand every year [26].

Adapting ways of working has also allowed for innovation and creativity. The demands of the pandemic and associated disruption have necessitated a close review of the programme. For example, due to a slot vacated by a session which could not be delivered online, it was suggested that a fortnightly ‘mentoring session’ from a former patient who shares her experience and offers insights to questions posed by the group would be useful. Although this might have been considered prior to the introduction of virtual working, this addition to the programme has been emphatically welcomed by both patients and staff.

## Conclusion

At a time in history where nearly every industry must adapt its way of functioning, the field of EDs is no exception. Online videoconferencing platforms have given the BEDS tools to continue as a functioning team, affording continuation of clinical care during unprecedented times. Whilst there are certain limitations to this way of working and increased risks of virtual working not covered in this paper, the team has found it possible to adapt an intensive programme for the treatment of individuals with EDs at high medical risk. Whilst the daypatient programme staff and patients agree that the virtual programme is no substitute for face-to-face treatment, there are aspects to this way of working which may outlast this pandemic.

Perhaps the key lesson learned is that it would have been possible to have accepted the status quo and adapted the service to manage, rather than exploit, the situation. However, the courage of the patients under the team’s care manifested in a resolve to continue the already difficult journey of recovery. The service was able to continue a positive and recovery-oriented culture, developing confidence and trust in the team during difficult circumstances. Perhaps, it is the *continuation* from a face-to-face programme which allowed the online programme to work. It would likely be more difficult to establish group coherence and a pro-recovery culture in an online programme with a ‘new’ patient group. Becoming a virtual daypatient programme has been possible because of this established recovery-oriented culture among patients as well as a collective sense of “We will find a way to make it work” rather than an assumption that it cannot be done, because it has not been done before. As other authors have highlighted, services need to adapt to the changing needs of patients and their families [10]. While it is too early to draw any conclusions regarding the long-term efficacy of the virtual daypatient programme, or indeed the impact of COVID-19 on clinical services or those suffering from EDs, it is hoped that this article can inform clinical care within EDs, and possibly beyond.

## Data Availability

Not applicable.

## Abbreviations

BEDS: Berkshire Eating Disorders Service
CBT: Cognitive Behaviour Therapy
ED: Eating disorder
EDE-Q: Eating Disorder Examination Questionnaire
PHQ9: 9-item Patient Health Questionnaire
GAD7: 7-item Generalized Anxiety Disorder questionnaire
